# Circulating Lineage 3 Recombination with NADC30-Like and NADC34-Like *Betaarterivirus suid* 2 in Taiwan

**DOI:** 10.1155/2024/6682052

**Published:** 2024-06-14

**Authors:** Chi-Fen Lee, Yen-Chen Chang, Hui-Wen Chang

**Affiliations:** ^1^ Graduate Institute of Molecular and Comparative Pathobiology School of Veterinary Medicine National Taiwan University TaipeiTaiwan; ^2^ School of Veterinary Medicine National Taiwan University TaipeiTaiwan

## Abstract

Porcine respiratory and reproductive syndrome (PRRS) caused by *Betaarterivirus suid* leads to severe economic losses. The emergence of highly pathogenic *Betaarterivirus suid* 2 (PRRSV-II), such as NADC30 and NADC34, has been reported in the USA and several Asian countries. NADC30-like PRRSV-II was first reported in 2018 in Taiwan. To investigate the PRRSV variants currently circulating in Taiwan, sequences covering ORF2-5 of Taiwan PRRSV isolates collected between 2020 and 2023 were analyzed. Phylogenetic analysis of the ORF5 nucleotide sequence indicated that most of the Taiwan isolates were clustered in lineage 3 and three isolates were grouped in lineage 1 and were closely related to the NADC34 strain. Interestingly, these three NADC34-like Taiwan PRRSV isolates carried amino acid deletions similar to NADC30 and were more closely related to NADC30 strains than the NADC34 strains in the Nsp2 gene. Next-generation sequencing and recombination detection program showed potential recombination of lineage 3 with NADC30- and NADC34-like PRRSV-II. Our results suggest the presence of circulating mosaic recombinants and lineage 3 PRRSV-II in Taiwan during 2020 and 2023.

## 1. Introduction

Porcine reproductive and respiratory syndrome (PRRS) is caused by *Betaarterivirus suid* (PRRSV), which belongs to the family Arteriviridae and the order of the Nidovirales. Members of this order are enveloped viruses that carry linear, positive-sense, and single-stranded RNA. The clinical manifestations of PRRSV infection in neonatal piglets to sows/boars vary from asymptomatic to devastating diseases [1], such as a lower food conversion ratio, respiratory and reproductive symptoms, and various mortalities [1]. Furthermore, PRRSV infection modulates host immunity, resulting in immune suppression and increased susceptibility to secondary infections in pigs [2].

The overall genome size of PRRSV is approximately 15 kb and comprises 11 open reading frames (ORFs) [3]. From ORF2 to ORF7, the sequences of the subgenomic mRNA templates overlapped with those of adjacent genes. The major envelope glycoproteins, including enveloped glycoprotein 5 (GP5), membrane protein (M), and nucleocapsid (N), encoded by ORF5, ORF6, and ORF7, respectively, are the most abundant glycoproteins in PRRSVs. Several investigations have shown that GP5 and M proteins are important immunogenic proteins that are critical for viral assembly [4, 5, 6, 7, 8], and that N protein forms a homodimer linked by disulfate bonds and plays a critical role in viral capsid formation [9]. Minor envelope glycoproteins, including GP2a, GP3, and GP4, which are responsible for viral receptor recognition, may also play important roles in immunogenicity [10, 11, 12, 13].

According to the International Committee on Taxonomy of Viruses classification and the WOAH Terrestrial Manual 2021, *Betaarterivirus suid* 1 (PRRSV-I) and *Betaarterivirus suid* 2 (PRRSV-II) are used to distinguish the subgenera *Eurobartevirus* and *Ampobartevirus*, respectively [14, 15]. Depending on different studies, PRRSV-I and PRRSV-II can be further classified into different subtypes or lineages [16, 17]. In Taiwan, previous studies indicated that most of the PRRSV isolates belong to PRRSV-II lineage 3 and some belong to lineage 1 and lineage 5 before 2019 [18, 19]. To date, only a few PRRSV-I vaccines and vaccine-like strains have been reported in Taiwan [20].

Several lineage 1 highly pathogenic PRRSV-II strains, such as MN184, NADC30, and NADC34 PRRSV-II strains, have been reported in the USA since 2002 causing severe abortion and death of fattening pigs regardless of vaccination status [18, 21, 22, 23]. MN184 and NADC30 PRRSV-II have a characteristic discontinuous 131-amino acid (aa) deletion in the nonstructure protein 2 (Nsp2) domain, whereas NADC34 PRRSV-II carries a discontinuous 100-aa deletion in the Nsp2 domain. In Taiwan, based on ORF5 and Nsp2 sequences, a NADC30-like PRRSV-II strain, which caused severe massive abortions, in 2018 was reported [18]. In China, a unique discontinuous 30-aa deletion in Nsp2 was also observed in a highly pathogenic PRRSV; however, this finding was unrelated to virulence [24, 25].

Phylogenetic analysis of ORF2-5 sequences of Taiwan PRRSV field isolates from 2020 to 2023 was performed to investigate the PRRSV variants currently circulating in Taiwan. Sequencing of Nsp2 gene was conducted to characterize the aa sequences of detected lineage 1 PRRSV-II strains based on ORF-5 gene. Next-generation sequencing revealed a lineage 3 PRRSV-II strain carrying the recombination with lineage 1 NADC30-like and NADC34-like PRRSV-II. The results suggest the presence of circulating lineage 3 PRRSV-II and its mosaic recombinant with NADC30-like and NADC34-like PRRSV-II in Taiwan from 2020 to 2023 and highlight the importance to develop vaccines against these Taiwan PRRSV-II variants for better controlling PRRS.

## 2. Materials and Methods

### 2.1. Sample Collection

A total of 136 PRRSV-II real-time reverse transcriptase PCR (RT-PCR)-positive serum and/or homogenized tissues from pigs with clinical symptoms (Table 1) or from health check collected during 2020–2023 from the Animal Disease Diagnostic Center (ADDC) Yunlin division at National Taiwan University were used in the study. The location of ADDC in Yunlin is advantageous because Yunlin is the largest pig-farming county in Taiwan and bears almost one-third of the total number of pigs in Taiwan. Most samples were collected from Yunlin County, except one sample was collected from a farm in Yilan County and another from Chiayi County.

### 2.2. Viral RNA Extraction and Reverse Transcription

The viral RNA was extracted using QIAamp® Viral RNA Mini Kit (Qiagen, Germany) and RNeasy Mini Kit (Qiagen, Germany) according to manufacturer's instruction. Reverse transcription was performed using the Transcriptor First Strand cDNA Synthesis Kit (Roche®, Switzerland) and the sequence-specific reverse primers ORF5R and Nsp2-nR, as listed in Table 2.

### 2.3. Polymerase Chain Reaction (PCR) Amplification and Sequencing

The PCR was conducted using AmaR OnePCR™ (GeneDireX, USA) reagent with the program including an initial 94°C for 5 min, followed by 35 cycles of denaturing at 94°C for 1 min, annealing at 55°C for ORF2-5 or at 52°C for Nsp2 for 1 min, and 72°C for 3 min. After a final extension at 72°C for 5 min, the PCR products were detected using a 1.5% EtBr-containing electrophoresis gel with UV illumination, and the amplicons were sent to Tri-I Biotech Inc. (Taipei, Taiwan) for Sanger sequencing. The RNA of sample NTUYL2020-133-S1 extracted using the method described above was directly sent to Tri-Biotech Inc. (Taipei, Taiwan) for next-generation sequencing (NGS).

### 2.4. Phylogenetic and Recombination Analysis

The sequences were aligned with published strains and analyzed using MEGA X software [28]. Phylogenetic trees based on different ORFs were generated using the maximum likelihood method and the Kimura 2-parameter model with 1,000 bootstrap replicates. Potential recombinant sites were estimated by RDP5 Beta5.54. Putative recombination sites were considered only when at least six out of nine of the methods, including EDP, GENECONV, BootSacn, MaxiChi, Chimaera, SiScan, 3Aeq, LARD, and PhyloPro, detect the recombination [29]. Distance plot and similarity plot were generated by using RDP and SimPlot version 3.5.1, respectively [30].

## 3. Results

### 3.1. Sample Collection and Sequencing Results

A total of 136 PRRSV-II positive samples were collected for this study. However, only 25 of them were successfully amplified using the primer pair of ORF2F and ORF5R, which targets the ORF2-ORF5. The resulting amplicon spans approximate 2.3 kb. Among these amplicons, 17 samples were successfully sequenced and eight samples were partially sequenced. These 25 samples were collected from 24 different farms. Twenty-two farms were located in Yunlin County, one was located in Chiayi County, and one was located in Yilan County. The sequences were uploaded in GenBank under accession number OR767241-OR767275 and OR837087-OR837088 and aligned with other PRRSV reference sequences from GenBank.

### 3.2. Sequence and aa Analysis of Different ORFs

**Figure 1:**
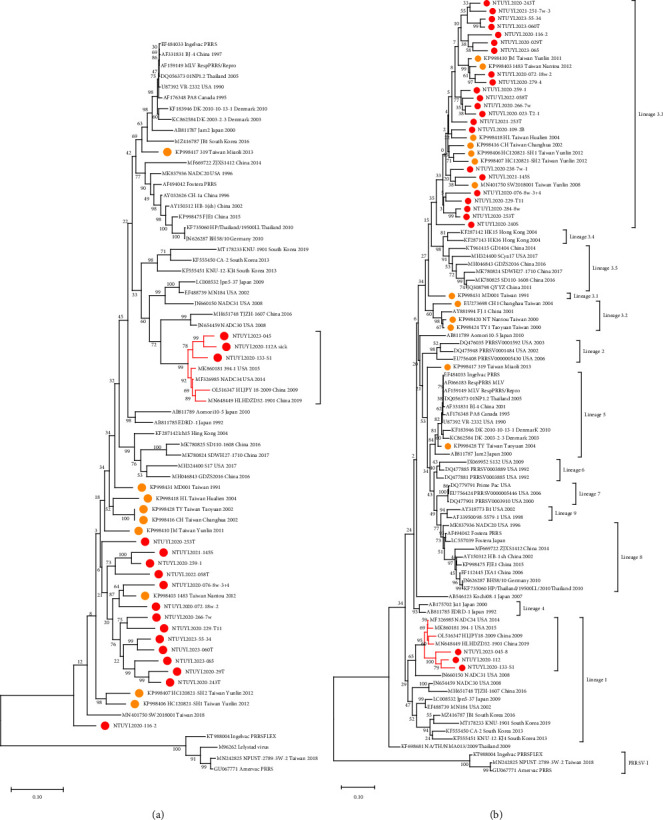
Phylogenetic tree of Taiwan PRRSV isolates and other historical strains based on ORF2-5 (a) and ORF5 (b). Maximum likelihood phylogenetic trees were constructed using Kimura 2-parameter model and tested with 1,000 bootstrap models. The Taiwan historical strains were labeled with orange dots, and the Taiwan isolates from 2020 to 2023 were labeled with red dots. Those without labeling were used as the reference historical strains from the GenBank database. (a) Regardless of which ORFs, NTUYL2020-112, NTUYL2020-133-S1, and NTUYL2023-45 were closely clustered with NADC34. (b) Based on ORF5 sequences, the Taiwan isolates were mainly clustered in lineage 3.3.

The nucleotide identities of sequences, covering ORF2 to ORF5, among the Taiwan isolates collected in the present study ranged from 80.23 to 94.38% (Table 3). The Taiwan isolates shared approximately 83%–85% nucleotide similarity with VR-2332 and PRRSV-II MLV strains (data not shown). Phylogenetic analysis based on ORF2-5 illustrated that most of the Taiwan isolates were closely grouped with Taiwan historical strains, except for NTUYL2020-112, NTUYL2020-133-S1, and NTUYL2023-45, which were grouped with the highly pathogenic PRRSV-II strain NADC34 [31] (Figure 1(a)). Phylogenetic analysis based on ORF5 nucleotide sequences revealed that all Taiwan isolates in this study belonged to PRRSV-II lineage 3.3, except for NTUYL2020-112, NTUYL2020-133-S1, and NTUYL2023-45, which belonged to lineage 1 (Figure 1(b))

### 3.3. Analysis of Deduced Amino Acid (aa) Sequences of GP2, GP2a (E Protein), GP3, GP4, and GP5

Amino acid substitutions in the previously published conserved B-cell and T-cell epitope domains of GP2, GP3, GP4, and GP5 of Taiwan PRRSV strains isolated in the present study are listed in Table 4. In the analysis of GP2a, the deduced aa sequences of GP2a in the Taiwan isolates were 255–256 aa. An aa deletion (175 aa) in the NTUYL2020-266-7w was noted in GP2a. In the analysis of GP2b (E protein), the deduced aa sequence of GP2b of all Taiwan isolates in this study contained 73 aa with sequence similarities, ranging from 86.84% to 100% (Table 3). Currently, there is no publication describing the immune epitopes of PRRSV GP2b. In the analysis of GP3, the deduced aa sequence of all Taiwan isolates in the present study contained 254 aa with sequence similarities about 68.84%–97.61% among each other (Table 3). Several aa substitutions were detected in the B- and T-cell epitopes of GP3 (Table 4). In the analysis of GP4, the deduced amino acid sequence of GP4 of the Taiwan isolates contained 176–178 aa. There was one Taiwan isolate, the NTUYL2020-0768w-3 + 4, exhibiting five aa deletions (91 aa and 97–100 aa), and three aa insertions (104–106 aa) compared to other Taiwan strains. All Taiwan isolates in this study had multiple substitutions in the B- and T-cell epitopes of GP4 (Table 4) [32, 36]. In the analysis of GP5, all Taiwan isolates in the present study were composed of 200 aa. The primary neutralizing epitope (PNE) was highly conserved among the Taiwan isolates, with 21/25 isolates possessing ^37^SYSQLIYNL^45^ and 1/25 possessing ^37^SYFQSIYNL^45^. Taiwan Lineage 1 isolates possessed different epitopes, ^37^FHLQLIYNL^45^, and ^37^FHLQSIYDL^45^, from other Taiwan isolates. All Taiwan isolates exhibited substitutions in the core aa of the PNE [32], at aa 38 from histidine (H) to tyrosine (Y), whereas this alteration was absent in the lineage 1 Taiwan isolates in the present study. The decoy B cell was highly conserved among the Taiwan isolates, with 15 and 3 out of 25 cases sharing identical sequences, VLVN and ALVN, respectively [33]. There were several substitutions in the T-cell epitope located at aa 145–164 of the conserved GP5 domain in all Taiwan isolates (Table 4) [36]. At aa 152, the original leucine (L) was switched to isoleucine (I) in all Taiwan isolates, except for lineage 1 Taiwan isolates. Lineage 1 Taiwan isolates possessed unique amino acid identities in the reported epitopes, where PNE revealed no substitution in the core amino acids and aa 152 remained in L, as in previous studies (Table 4) [6, 33, 36, 37]. In addition to the documented epitope, GP5 deduced protein sequence from 27 to 61 aa could be used to further subdivide lineage 1 into several sublineages [38]. According to the 50% consensus sequence template, the Taiwan lineage 1 isolates were mostly likely to be classified as the L1A sublineage.

### 3.4. Nsp2 Nucleotide and Amino Acid Analyses

**Figure 2:**
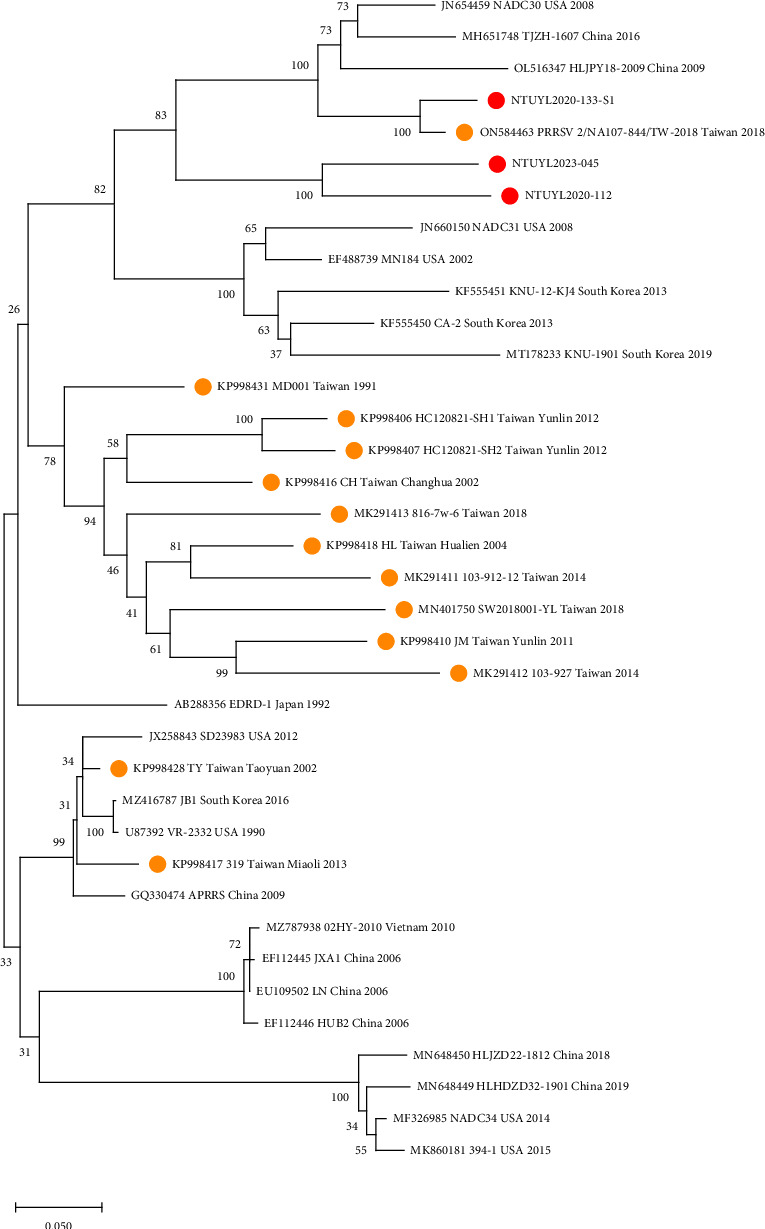
Phylogenetic tree of PRRSV based on Nsp2 gene. A maximum likelihood phylogenetic tree was constructed using Kimura 2-parameter model and tested with 1,000 bootstrap models. The Taiwan historical strains were labeled with orange dots, while the Taiwan isolates from 2020 to 2023 were labeled with red dots. Those without labels were used as the reference historical strains from the GenBank database. Lineage 1 Taiwan isolates, NTUYL20202-112, NTUYL2020-133-S1, and NTUYL2023-45, were closely clustered with NADC30 and a lineage 1 isolate from Taiwan in 2018.

**Figure 3:**
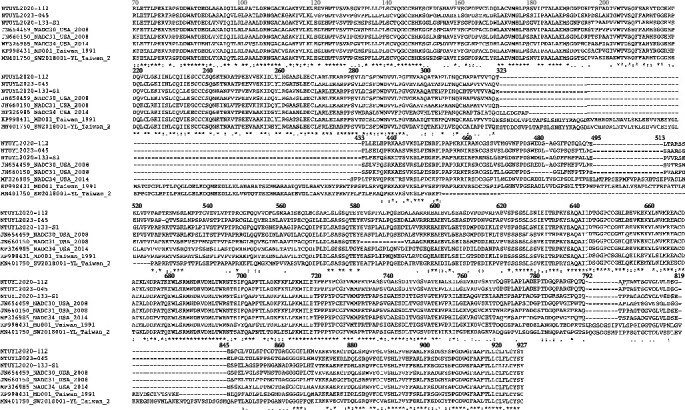
Amino acid sequence alignment of partial Nsp2 gene. Three Taiwan PRRSV isolates displayed similar amino acid deletion patterns as NADC30. Asterisk ( ^*∗*^) represents 100% identity of a single residue. Colon (:) represents a strong conserved group with >0.5 under Gonnet Pam250 matrix. Dot (.) represents a weakly conserved group with <0.5 under Gonnet Pam250 matrix. Dash (-) indicates amino acid not available. MD001 (PK998431) is the first isolated PRRSV in Taiwan in 1991, while SW2018001 (MN401750) was isolated in the year 2018.

The partial Nsp2 nucleotide sequences and deduced aa sequences were aligned. Interestingly, the nucleotide sequences of the three Taiwan lineage 1 isolates were more closely related to NADC30 than to NADC34 (Figure 2 and Table 5). Importantly, all three isolates displayed identical deletions as in NADC30, including a large 131-discontinuous aa deletion from aa to 323–433, aa 482, and aa 495–513 while using the PRRSV-II prototype-VR2332 as the template (Figure 3).

### 3.5. Next-Generation Sequencing and Recombination Analysis

**Figure 4:**
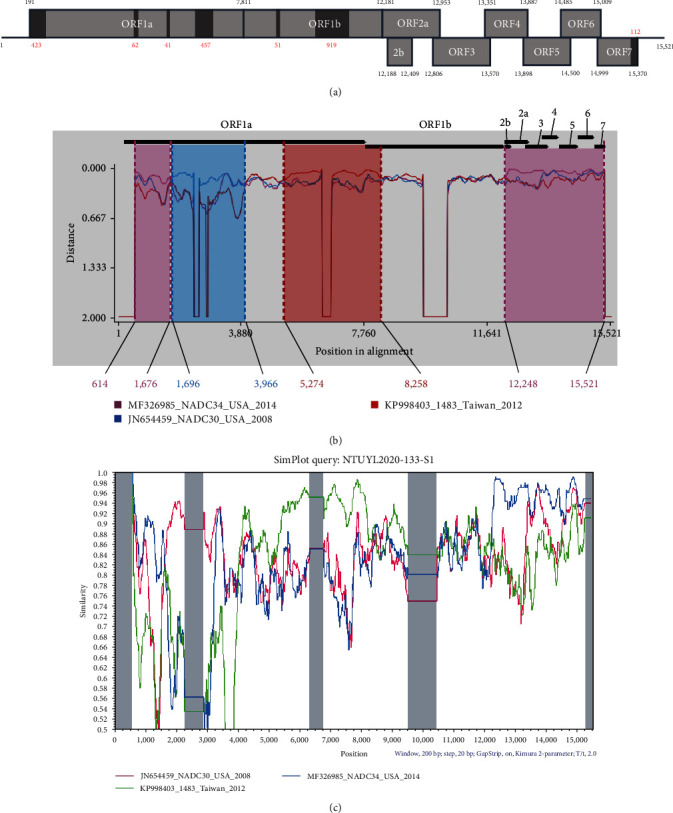
NTUYL2020-133-S1 NGS result spanning region, distance plot, and similarity plot compared to NADC30 and NADC34. (a) The genome of PRRSV. The dark gray regions revealed the missing nucleotide in NGS. The black Arabic numerals annotate the nucleotide number while using MD001 (PK998431) as the template. The red Arabic numerals annotate the total number of missing nucleotide in the light gray regions. (b) DNA distance plot while using NTUYL2020-133-S1 as the potential recombinant strain and comparing it to NADC30, NADC34, and 1483. Potential recombination breakpoints are annotated by different colors. Light lavender annotates potential recombinant regions with NADC34; light blue annotates potential recombinant regions with NADC30; and light red annotates potential recombinant regions with 1483. (c) Similarity plot while using NTUYL2020-133-S1 as the query. The light gray region annotated the missing nucleotides by NGS.

Approximately 43 million readings were collected from the NGS results. Among all the readings, there were 1649 readings correlating to the reference strains, NADC34 (MF326985) and NADC30 (JN654459), which spanned partial ORF1a, ORF1b, and ORF7 and complete ORF2−6 (Figure 4(a)). Using MD001 (PK998431) as the template, NTUYL2020-133-S1 covered nucleotide position 614–4123, 4186–5462, 5504–6343, 7811–8813, 8865–9524, and 10444–15258 (Figure 4(a)).

Using NTUYL2020-133-S1 as the query and NADC30, NADC34, MN184, 1483, SW2018001, and MD001 as the reference strains, four potential breakpoints located at nucleotide position 1–1676/12248–15521, 1696–3966, 5274–8258, and 8910–10457 (event 1–4) were detected (Table 6). These potential recombination signals were detected positively using at least six different recombination detection methods. Event 1 demonstrated that NTUYL2020-133-S1 potentially recombined with NADC34 in the early ORF1a region and ORF2-7 regions. This recombination breakpoint contained 4,687 nucleotides and had the highest nucleotide identity 93.7% with NADC34. A phylogenetic tree was constructed based on the 4678-nucleotide sequence, showing that NTUYL2020-133-S1 and NADC34 were grouped together (Figure S1). Event 2 demonstrated that the NTUYL2020-133-S1 potentially recombined with NADC30 in the hypervariable region of ORF1a region. This recombination breakpoint contained 2,271 nucleotides and had the highest nucleotide identity 88.2% with NADC30. A phylogenetic tree was constructed based on the 2271-nucleotide sequence, showing that NTUYL2020-133-S1 and NADC30 were grouped together (Figure S1). Event 3 demonstrated NTUYL2020-133-S1 potentially recombined with 1483 (KP998403) in the late ORF1a and early ORF1b regions. This recombination breakpoint contained 2,985 nucleotides and had the highest nucleotide identity 94.3%–1483. A phylogenetic tree was constructed based on the 2985-nucleotide sequence, showing that NTUYL2020-133-S1 and 1483 were grouped together (Figure S1). Event 4 demonstrated NTUYL2020-133-S1 potentially recombined with SW2018001 (MN401750) in ORF1b region. This recombination breakpoint contained 1548 nucleotides and had the highest nucleotide identity 87.9% with SW2018001. A phylogenetic tree was constructed based on the 1548-nucleotide sequence, showing that NTUYL2020-133-S1 and SW2018001 were grouped together (Figure S1). However, in event 4, there was a 919-nucleotide missing in the potential event; therefore, we excluded this data in the later distance plot. Distance plot and similarity plots visualized the geographic distribution of the potential recombinant breakpoints (Figures 4(b) and 4(c)).

We further performed recombination detection based on ORF2-5 nucleotide sequences in 25 Taiwan isolates and other reference strains from GenBank. A putative recombination event was noted in NTUYL2020-072-18w-2 from the 3′ terminal of ORF3 to the 3′ end of ORF4. This recombination breakpoint contained 215 nucleotides and shared the highest nucleotide identity, 97.6%, to 934-1 (MK860181). 934-1 is grouped with NADC34 based on the ORF2-5 nucleotide sequence. A phylogenetic tree was constructed based on the 215-nucleotide sequence, showing that NTUYL2020-072-18w-2, 934-1, and NADC34 were grouped together and were closely related to the lineage 1 isolates from Taiwan (Figure S1).

## 4. Discussion

In the swine industry, PRRSV has devastating impacts and pressure on pigs of all ages because of its variable clinical presentations and the lack of fully protective vaccines. In the present study, we amplified and sequenced PRRSV genomes covering complete or partial ORF2–ORF5. Our results suggest that the major PRRSV strains currently circulating in Taiwan from 2020 to the mid-2023 belong to PRRSV-II, lineage 3.3. This finding is similar to that of a previous epidemiological study conducted in Taiwan from 1991 to 2013 [19]. Furthermore, we also speculated that there were circulating recombinants of NADC30-like and NADC34-like lineage 1 strains with lineage 3.3 PRRSV-II in Taiwan based on the identification of three Taiwan PRRSV-II lineage 1 strains exhibiting high sequence identity to NADC34 strain in ORF2-5, the detection of unique aa deletions identical to NADC30, and the high sequence identity to NADC30 strain in Nsp2 gene. We further sequenced one of the isolates NTUYL2020-133-S1 using NGS. Although part of the genome was missing due to insufficient RNA concentration in the sample, most of the important genome segments were successfully sequenced. We used RDP Beta 5.54 to validate the finding. RDP results revealed four potential breakpoints in NTUYL2020-133-S1 with NADC34, NADC30, 1483, and SW2018001. Because of the missing nucleotide sequences in event 4, we excluded potential recombination with SW2018001 in our study. The distance plot and similarity plot also showed potential breakpoints of NTUYL2020-133-S1. To the best of our knowledge, this is the first study reporting recombinant NADC30/34/1483-like strains in Taiwan. Combined with the identification of several aa substitution(s) spanning documented immunogenic epitopes in GP2a, GP3, GP4, and GP5 of Taiwan strains; this suggests that Taiwan PRRSV strains may have evolved distinctly owing to geographic isolation, but Taiwan pig-farming still faces the risk of importing of other PRRSV lineages and potential recombination events.

PRRSV NADC30 was first detected in the USA in 2008 and has spread throughout China since 2015 [21, 39]. The emergence of a PRRSV-II lineage 1 NADC30-like strain was reported in Taiwan in 2018, and the appearance of this specific lineage is speculated to have been imported from the USA [18]. PRRSV NADC34 was first detected in the USA in 2014 and has affected China since 2017 [31, 40]. In Peru and South Korea, NADC34-like PRRSV has been detected in 2015 and 2022, respectively [41, 42]. The South Korean strain has been demonstrated as the recombinant of a NADC34-like PRRSV-II with the MLV vaccine strain [42]. 1483 PRRSV-II strain is a Taiwan historical strain and belongs to lineage 3 as mentioned in an epidemiological study [19]. However, the pathogenicity and its clinical effects on swine are unknown. In the present study, the presence of lineage 1 and 3 recombinant PRRSV-II NADC30/34/1483-like viruses suggests the potential import of NADC34-like and NADC30-like or recombinant NADC30/34-like strains into Taiwan since 2020. Since PRRSV-II lineage 3 is only restricted to parts of East Asia, the recombination event of lineage 3 between other lineages is also limited. Till now, lineage 3 strains have been reported to recombine with lineages 1 and 8 [43, 44, 45, 46, 47, 48, 49]. At the sublineage level, we mentioned earlier that Taiwan lineage 1 isolates belong to the L1A sublineage based on ORF5 aa sequence, as well as NADC34. In contrast, NADC30 belongs to L1C sublineage. Pamornchainavakul's study (2022) indicated that intralineage recombination is more frequent than the interlineage recombination [50]. Furthermore, their result showed that recombination event between L1A and L1C seemed to occur more often in ORF1a. A similar recombination pattern could be found in different continents, in the USA and East Asia, which also correlates with our findings. Interestingly, the Taiwan NADC30/34/1483-like isolates shared identical deletion patterns of Nsp2 with one of the Taiwan 2018 isolates, NA107-844 (ON584463), which was reported as an NADC30-like strain back in 2018 in Taiwan [18]. The Nsp2 nucleotide and aa similarity were up to 92% and 88%, respectively (data not shown). The ORF2-5 nucleotide sequence of NA107-844 was closely related to NADC34. The result may suggest that Taiwan NADC30/34-like strains might have originated in 2018. Further investigations and monitoring of PRRSV epidemiology should be conducted to understand the route of viral transmission and prevent the transboundary reemerging disease.

Due to client confidentiality and most of the samples (serum and tissues) were collected and submitted by swine veterinarians from animal vaccine/drug companies to our ADDC with limited information of history, we are unfortunately not able to provide contextualizing data to further interpret the pathogenicity of different variant PRRSV-II on different managements or coinfection status. However, we believe that the epidemiology data of our first revealing novel PRRSV-II variants exhibiting multiple recombination events of lineage 1 and 3 NADC30/34/1483-like PRRSVs and the updated information of circulating PRRSV-II lineage 3.3 in Taiwan are important for developing new vaccines and governmental interventions in controlling the disease. In addition to these recombinant PRRSVs, the main species, PRRSV-II, displayed unique substitutions in B- or T-cell epitopes. By combining Taiwan and global analysis data, potential functions of these epitopes should be further investigated for inventing better prevention methods or vaccines in the future.

## 5. Conclusion

This study highlights the dominant PRRSV-II lineage 3.3 circulating during 2020–2023 in Taiwan and the first identification of lineages 1 and 3 recombinant NADC30/34/1483-like PRRSV since 2020. The PRRSV-II Taiwan isolates carrying unique epitopes may indicate the necessity of understanding their evolutionary advantage while against the host immunity for inventing better prevention methods or vaccines in the future.

## Figures and Tables

**Table 1:** Sample details in this study.

Viral ID	County	Year of isolation	Sample/no. of pig collected	PRRSV species	Lineage^a^	Clinical signs	Accession number
NTUYL2020-023-T2-1	Yunlin	2020	Lung/1	II	Lineage 3.3	Panting and diarrhea with 40% morbidity and 100% fatality	OR767264 OR767269
NTUYL2020-029T	Yunlin	2020	Tissue mix/4	II	Lineage 3.3	Health check	OR767247
NTUYL2020-072-18w-2	Yunlin	2020	Serum/1	II	Lineage 3.3	Clinically PDNS^b^ and panting	OR767244
NTUYL2020-076-8w-3 + 4	Yunlin	2020	Serum/2	II	Lineage 3.3	Health check	OR767245
NTUYL2020-109-2B	Yunlin	2020	Lung/1	II	Lineage 3.3	Clinically panting	OR837087
NTUYL2020-112	Yunlin	2020	Serum/1	II	Lineage 1	Not provided	OR767248 OR767274
NTUYL2020-116-2	Yunlin	2020	Tissue mix/1	II	Lineage 3.3	Clinically panting with 100% morbidity and 30% fatality	OR767249
NTUYL2020-133-S1	Yunlin	2020	Serum/1	II	Lineage 1	Not provided	OR767243 OR767275 PP647750 (NGS)
NTUYL2020-229-T11	Yunlin	2020	Tissue mix/3	II	Lineage 3.3	High fever and respiratory signs	OR767251 OR767270
NTUYL2020-238-7w-1	Yunlin	2020	Serum/1	II	Lineage 3.3	Clinically healthy	OR767265 OR767271
NTUYL2020-240S	Yilan	2020	Serum/3	II	Lineage 3.3	High fever and respiratory signs	OR767260 OR767267
NTUYL2020-243T	Yunlin	2020	Tissue mix/1	II	Lineage 3.3	Not provided	OR767252
NTUYL2020-253T	Yunlin	2020	Tissue mix/>1	II	Lineage 3.3	Lathery and respiratory signs	OR767253
NTUYL2020-259-1	Yunlin	2020	Tissue mix/1	II	Lineage 3.3	Respiratory sign and high fatality rate in weaning pigs	OR767254
NTUYL2020-266-7w	Yunlin	2020	Serum/9	II	Lineage 3.3	Respiratory signs with 10%–20% morbidity	OR767255
NTUYL2020-279-4	Yunlin	2020	Serum/1	II	Lineage 3.3	Not provided	OR767272
NTUYL2020-284-8w	Yunlin	2020	Serum/>2	II	Lineage 3.3	Not provided	OR767261 OR767268
NTUYL2020-301-6w	Yunlin	2020	Serum/4	II	Not applicable	Not provided	OR767262
NTUYL2021-145S	Yunlin	2021	Serum/1	II	Lineage 3.3	Not provided	OR767256
NTUYL2021-253T	Yunlin	2021	Tissue mix/1	II	Lineage 3.3	Not provided	OR837088
NTUYL2022-058T	Yunlin	2022	Tissue mix/1	II	Lineage 3.3	Lethargy, subcutaneous edema, and diarrhea with 10%–20% morbidity and 5% fatality	OR767257
NTUYL2023-45	Chiayi	2023	Serum/1	II	Lineage 1	Abortion storm in sow	OR767258 OR767273
NTUYL2023-055-34	Yunlin	2023	Serum/2	II	Lineage 3.3	Weaning pig diarrhea and sow abortion	OR767259
NTUYL2023-060T	Yunlin	2023	Tissue mix/1	II	Lineage 3.3	Panting and arthritis with 10% morbidity and 50% fatality	OR767242
NTUYL2023-065	Yunlin	2023	Tissue mix/3	II	Lineage 3.3	Respiratory signs, arthritis with 20% fatality	OR767241

^a^Lineage, lineage was defined based on ORF5 phylogenetic analysis. ^b^PDNS, porcine dermatitis nephropathy syndrome.

**Table 2:** Primer pairs used in this study.

Primer code	Primer sequences (5′ to 3′)	Product size (bp)	Position^a^	References
ORF2F	ATT ACA ATG ATG CGT TTC GTG CG	—	12,075–12,097	This study
ORF5R	CGG CCG CGA CTC ACC TTT AG	2,541	14,596–14,615	[26]
Seq 1	CAG GGT CAA ATG TAA CCA TA	—	12,718–12,737	This study
Seq 2	CTG CAA GCC ATG TTT CAG TTC	—	13,428–13,448	This study
Nsp2-nF	GAA GGG AAT TGT GGT TGG CA	—	1,300–1,319	[27]
Nsp2-nR	AGA CCC AGA AAA CAC ACC CA	2,266–2,767	3,917– 3,936	[27]

^a^Strain MD001 (KP998431), the first isolated Taiwan PRRSV strain, was used as a template.

**Table 3:** Nucleotide and amino acid similarity among the Taiwan isolates and between the Taiwan isolates and VR-2332.

ORF	Nucleotide similarity (%)			Structural protein	Amino acid similarity (%)	
	VR-2332^a^	Taiwan isolates	*N*		VR-2332	Taiwan isolates
ORF2-5	83.24–87.11	80.23–94.38	17	—	—	—
ORF2a	85.11–89.03	82.10–100	22	GP2a	83.47–87.72	78.75–100
ORF2b	86.49–92.34	84.28–100	22	GP2b (E)	88.39–95.81	86.84–100
ORF3	85.48–88.07	82.02–97.61	16	GP3	77.09–83.79	68.84–97.61
ORF4	84.51–87.77	80.42–96.55	16	GP4	82.89–90.58	83.55–95.98
ORF5	81.29–85.84	75.61–97.29	25	GP5	77.69–85.50	74.51–97.29

^a^VR-2332 (U87392): PRRSV-II prototype.

**Table 4:** Antigenic epitopes of structural proteins, GP2a, GP3, GP4, and GP5, in PRRSV-II.

Viral proteins (total number of sequences in this study)	Epitope type	Sequence	aa position	References/strain	Sequence of the epitope (number of strains carrying the epitope sequence in this study)
GP2a (*N* = 22)	B-cell epitope	LPSLAGWWSSASDWF	41–55	[32]/NVSL 97–7,895 strain	SPLPDGWWSFVSDWF (12) SQSPVGWLSFASDWF (3) SQLPDGWWSFVSDWF (2) SLLPDGWWSFVSDWF (2) SQLPGGWWSFVSDWF (1) SPSPDGWWSFVSDWF (1) LQLPDGWWSFVSDWF (1)
	B-cell epitope	QAAWKQVVSEAT	124–135	[32, 33]/NVSL 97–7,895 strain; IA strain 97–7,895	**QAAWKQVVSEAT (10)** QAAWKQVVTEAT (8) QAAWRQVVTEAT (3) QAAWRQVVSEAT (1)
	B-cell epitope	TPGSRPKLHDFQ	196–207	[34]/BJ-4 strain	**TPGSRPKLHDFQ (11)** TSGSRPKLHDFQ (2) TPGSRPKLYNFQ (2) TPGSRPQLHDFQ (1) TPDSRPKLHDFQ (1) TPNSRPKLHDFQ (1) TPGSRPKLQDFQ (1) TPSSRPKLHDFQ (1) TPESRPKLHDFQ (1) TPGSRPKLQDFR (1)

GP3 (*N* = 16)	B-cell epitope	YEPGRSLW	68–74	[32, 35]/NVSL 97–7,895 strain; CH-1a strain	YEPSTSLW (6) YEPNRSLW (5) **YEPGRSLW (3)** YEPNRHLW (1) YEPSRSLW (1)
	B-cell epitope	WCRIGHDRCGED	74–85	[32, 35]/NVSL 97–7,895 strain; CH-1a strain	WCRIGQDRCTEA (3) WCRIGHDRCEEN (3) WCRIGQDRCTEN (2) WCRIGHDRCEEG (1) WCRIGHDRCEEA (2) WCKIGNDRCSEN (1) WCRIGNDRCGED (1) WCRIGQDRCTES (1) WCRIGNDRCSEG (1) WCKIGQDRCAED (1)
	B-cell epitope	RCSEDDHDDLGFMVP	81–95	[32]/NVSL 97–7,895 strain	RCTEADHDELGFVVP (2) RCEEADHDELGFMVP (2) RCTENDHDELGFMVP (2) RCEENDHDELGFMVP (2) RCEEGDHDELGFVVP (1) RCSENDRDELGFMVP (1) RCGEDDHDELGFFVP (1) RCTESDHDELGFMVP (1) RCSEGDHDGAGVYGA (1) RCAEDDHDELGFVVP (1) RCEENDHDELGFVVP (1) RCTEADHDDLGFVVP (1)
	B-cell epitope	GFMVPPGLSSEGHLT	95–105	[32]/NVSL 97–7,895 strain	**GFMVPPGLSSEGHLT (6)** GFVVPPGLSSEGHLT (4) GFMVPPGLSNEGHLT (2) GFVVPPGLSIEGHLT (1) GFFVPPGLSNESHLT (1) GVYGAARSLSEGHLT (1) GFVVPPGLSSEGHLS (1)

GP4 (*N* = 16)	B-cell epitope	SCLRHGDSSSQTIRK	51–65	[32]/NVSL 97–7,895 strain	SCLRHGDPSGKTFRK (3) SCLRHGNTTSAAFSK (1) SCLRHGNTSSEAFRK (1) SCLRHGNTTTETFRK (1) SCSGHGNTTSQTFRK (1) SCLRHGNTTSETFRK (1) SCFGHGNTSSETSRK (1) SCLRHGNTTSKAFSK (1) SCLRYGNPSSAGLRK (1) SCLRHGNTASEAFRK (1) SCLRHDNPSSATFRK (1) SCFRHGNPSSATFRK (1) SCLRHGNTTSEAFRK (1) SCLRHGNTTNKAFRK (1)
	T-cell epitope	QHVREFTQRSLMVDHVRLLH	131–150	[36]/SD23983 strain	QHVKEFTQRSLVVDHVRLLH (7) QHVREVTQRSLVVDHVRLLH (1) QHVKEFTQRSLVIDHVRLLH (1) QHVKEVTQRSLVVDHVRLLH (1) QHVREFTQRSLVVDHVRLLH (1) QHVKEFTRRSLVVDHVRLLH (1) QHVKEFTRRSLMVDHVRLLH (1) QHVKEFTQRSLVVDHVRVLQ (1) QHVKEITQRSLVVDHVRLLH (1) QHVKEFTQRSLVVDHVRLVH (1)

GP5 (*N* = 25)	B-cell epitope ^*∗*^, a (PNE)^a^	SHLQLIYNL^b^	37–45	[6, 33, 37]/IA strain 97–7895; VR-2,332 strain	SYSQLIYNL (21) FHLQLIYNL (2) SYFQSIYNL (1) FHLQSIYDL (1)
	B-cell epitope, decoy	A/(V)LVN	27–30	[33]/IA strain 97–7,895	**VLVN (15)/ALVN (3)** VPVN (2) ALVS (1) ALAS (1) VLVD (1) VLAG (1) AVVN (1)
	T-cell epitope	LLDTKGRLYRWRSPVIIEKR	145–164	[36]/SD23983 strain	LLDTKGRIYRWRSPVIIEKK (9) LLDTKGRIYRWRSPVIIERQ (3) LLDTKGRIYRWRSPVIVEKK (2) LMDTKGRIYRWRSPVIIEKK (1) LLDTKGRIYRWRSPVIIEKG (1) LLDTKGKIYRWRSPVIIEKN (1) LLDTKGRIYRWRSPIIIEKK (1) LQDTKGRIYRWRSPVIIEKK (1) LLDTKGRIYRWRSPVIIEKN (1) LLDTKGKIYRWRSPVIIEKK (1) LLDTKGRIYRWRSPVIIERK (1) FLDTKGQLYRWRFPVIIEKG (1) FLDTKGKLYRWRFSVIIEKG (1) LLDPKGQLYRWRFFVIIEKG (1)

The neutralizing epitope is marked with a  ^*∗*^. The bold highlights the same sequence as well-documented epitopes in the Taiwan isolates. ^a^PNE, Primary neutralizing epitope. ^b^Core amino acids are labeled with underline.

**Table 5:** Nucleotide similarity (%) of different ORFs between Taiwan lineage 1 isolates and historical strains.

Taiwan isolates	Historical strains	Nsp2	ORF2a	ORF2b	ORF3	ORF4	ORF5	ORF6
NTUYL2020-112	VR-2332	64.54	86.31	87.56	86.14	84.73	81.00	—
	NADC30	**73.83**	82.77	86.95	86.71	92.75	84.17	—
	NADC34	65.26	**94.60**	**95.32**	**95.28**	**94.36**	**92.10**	—
	MD001	61.78	85.78	89.63	87.10	84.36	79.03	—
	SW2018001	57.58	83.74	89.20	85.76	82.84	79.79	—

NTUYL2020-133-S1	VR-2332	67.96	85.39	86.49	85.26	85.21	81.29	89.27
	NADC30	**80.80**	81.95	84.71	85.99	93.78	82.59	92.86
	NADC34	67.77	**95.31**	**94.36**	**96.26**	**94.98**	**91.11**	**95.02**
	MD001	64.78	85.83	88.62	85.87	84.38	78.44	89.53
	SW2018001	59.76	82.45	87.05	83.84	81.44	80.95	90.44

NTUYL2023-45	VR-2332	66.90	85.52	86.49	87.44	86.60	80.74	—
	NADC30	**76.13**	82.10	85.86	87.56	94.99	85.06	—
	NADC34	66.22	**93.62**	**94.36**	**96.48**	**96.96**	**91.25**	**—**
	MD001	64.51	84.98	88.58	87.53	86.68	80.92	—
	SW2018001	60.07	84.24	89.20	86.08	84.02	79.77	—

MD001 (PK998431) is the first isolated PRRSV in Taiwan in 1991, while SW2018001 (MN401750) was isolated in year 2018. Only partial Nsp2 gene was included in this similarity matrix. The bold characters highlight the highest nucleotide similarity among different ORFs.

**Table 6:** RDP5 detected potential recombinant events and breakpoints in NTUYL2020-133-S1 and NTUYL2020-072-18w-2.

Event	Recombinant sequence	Breakpoint position	Parental sequence	*P* value						
		Start/end	Major/minor	RDP	GENECONV	Bootscan	Maxchi	Chimera	SiSscan	3Seq
1	NTUYL2020-133-S1	1/1676	1483/NADC34	1.986E-69	NA	3.082E-55	3.000E-200	2.232E-40	1.597E-39	1.699E-73
		12248/15521								
2	NTUYL2020-133-S1	1696/3966	1483/NADC30	9.699E-48	NA	2.684E-26	1.330E-31	2.854E-35	1.239E-25	1.642E-51
3	NTUYL2020-133-S1	5274/8258	Unknown/1483	1.707E-16	5.306E-6	5.853E-9	2.352E-17	3.118E-16	2.451E-37	1.343E-3
4	NTUYL2020-133-S1	8910/10457	Unknown/SW2018001	9.535E-4	NA	1.245E-4	3.789E-5	7.232E-5	6.728E-12	1.886E-2
5	NTUYL2020-072-18w-2	13643/13863	NTUYL2020-229-8/394-1	1.678E-8	1.576E-6	1.749E-5	6.505E-6	3.690E-2	1.543E-4	8.138E-4

NADC30 (JN654459), NADC34 (MF326985), 1483 (KP998403), SW2018001 (MN401750), and 394-1 (MK860181).

## Data Availability

The datasets generated and/or analyzed during the current study are not publicly available due to the privacy of the farms used in the study but are available from the corresponding author on reasonable request.

## Supplementary Materials

Supplementary Materials Figure S1: trees for potential recombination events. (A) Based on the 4687-nucleotide from nucleotide position 1-1676/12248-15521, NTUYL2020-133-S1 was grouped with NADC34. (B) Based on the 2271-nucleotide from nucleotide position 1696-3966, NTUYL2020-133-S1 was grouped with NADC30. (C) Based on the 2985-nucleotide from nucleotide position 5274-8258, NTUYL2020-133-S1 was grouped with 1483. (D) Based on the 1548-nucleotide from nucleotide position 8910-10457, NTUYL2020-133-S1 was grouped closely with SW2018001. (E) Based on the 215-nucleotide sequence, NTUYL2020-072-18W-2 was grouped with 934-1.
