# Supplementary material for: Circulating Lineage 3 Recombination with NADC30-Like and NADC34-Like Betaarterivirus suid 2 in Taiwan
Source: Transbound Emerg Dis. 2024 Jun 14;2024:6682052. doi: 10.1155/2024/6682052 (PMC12019867; doi:10.1155/2024/6682052)
Supplement: Supplementary Materials — Figure S1: trees for potential recombination events. (A) Based on the 4687-nucleotide from nucleotide position 1-1676/12248-15521, NTUYL2020-133-S1 was grouped with NADC34. (B) Based on the 2271-nucleotide from nucleotide position 1696-3966, NTUYL2020-133-S1 was grouped with NADC30. (C) Based on the 2985-nucleotide from nucleotide position 5274-8258, NTUYL2020-133-S1 was grouped with 1483. (D) Based on the 1548-nucleotide from nucleotide position 8910-10457, NTUYL2020-133-S1 was grouped closely with SW2018001. (E) Based on the 215-nucleotide sequence, NTUYL2020-072-18W-2 was grouped with 934-1. [file 6682052.f1.pdf]

Next-generation sequencing and recombination analysis

Approximately 43 million readings were collected from the NGS results. Using NTUYL2020-133-S1 as the query and NADC30, NADC34, MN184, 1483, SW2018001, and MD001 as the reference strains, four potential breakpoints located at nucleotide position 1-1676/12248-15521, 1696-3966, 5274-8258, and 8910-10457 (event 1 to 4) were detected. These potential recombination signals were detected positively using at least six different recombination detection methods. Event 1 demonstrated that NTUYL2020-133-S1 potentially recombined with NADC34 in the early ORF1a region and ORF2-7 regions. This recombination breakpoint contained 4687 nucleotides and had the highest nucleotide identity 93.7% with NADC34. A phylogenetic tree was constructed based on the 4678-nucleotide sequence, showing that NTUYL2020-133-S1 and NADC34 were grouped together (Supplementary Fig. S1A). Event 2 demonstrated that the NTUYL2020-133-S1 potentially recombined with NADC30 in the hypervariable region of ORF1a region. This recombination breakpoint contained 2271 nucleotides and had the highest nucleotide identity 88.2% with NADC30. A phylogenetic tree was constructed based on the 2271-nucleotide sequence, showing that NTUYL2020-133-S1 and NADC30 were grouped together (Supplementary Fig. S1B). Event 3 demonstrated NTUYL2020-133-S1 potentially recombined with 1483 (KP998403) in

the late ORF1a and early ORF1b regions. This recombination breakpoint contained 2985 nucleotides and had the highest nucleotide identity 94.3% to 1483. A phylogenetic tree was constructed based on the 2985-nucleotide sequence, showing that NTUYL2020-133-S1 and 1483 were grouped together (Supplementary Fig. S1C). Event 4 demonstrated NTUYL2020-133-S1 potentially recombined with SW2018001 (MN401750) in ORF1b region. This recombination breakpoint contained 1548 nucleotides and had the highest nucleotide identity 87.9% with SW2018001. A phylogenetic tree was constructed based on the 1548-nucleotide sequence, showing that NTUYL2020-133-S1 and SW2018001 were grouped together (Supplementary Fig. S1D). However, in event 4, there was a 919-nucleotide missing in the potential event. We further performed recombination detection based on ORF2-5 nucleotide sequences in 25 Taiwan isolates and other reference strains from GenBank. A putative recombination event was noted in NTUYL2020-072-18w-2 from the 3’ terminal of ORF3 to the 3’ end of ORF4. This recombination breakpoint contained 215 nucleotides and shared the highest nucleotide identity, 97.6%, to 934-1 (MK860181). 934-1 is grouped with NADC34 based on the ORF2-5 nucleotide sequence. A phylogenetic tree was constructed based on the 215-nucleotide sequence, showing that NTUYL2020-072-18w-2, 934-1, and NADC34 were grouped together and were closely related to the lineage 1 isolates from Taiwan (Supplementary Fig. S1E).

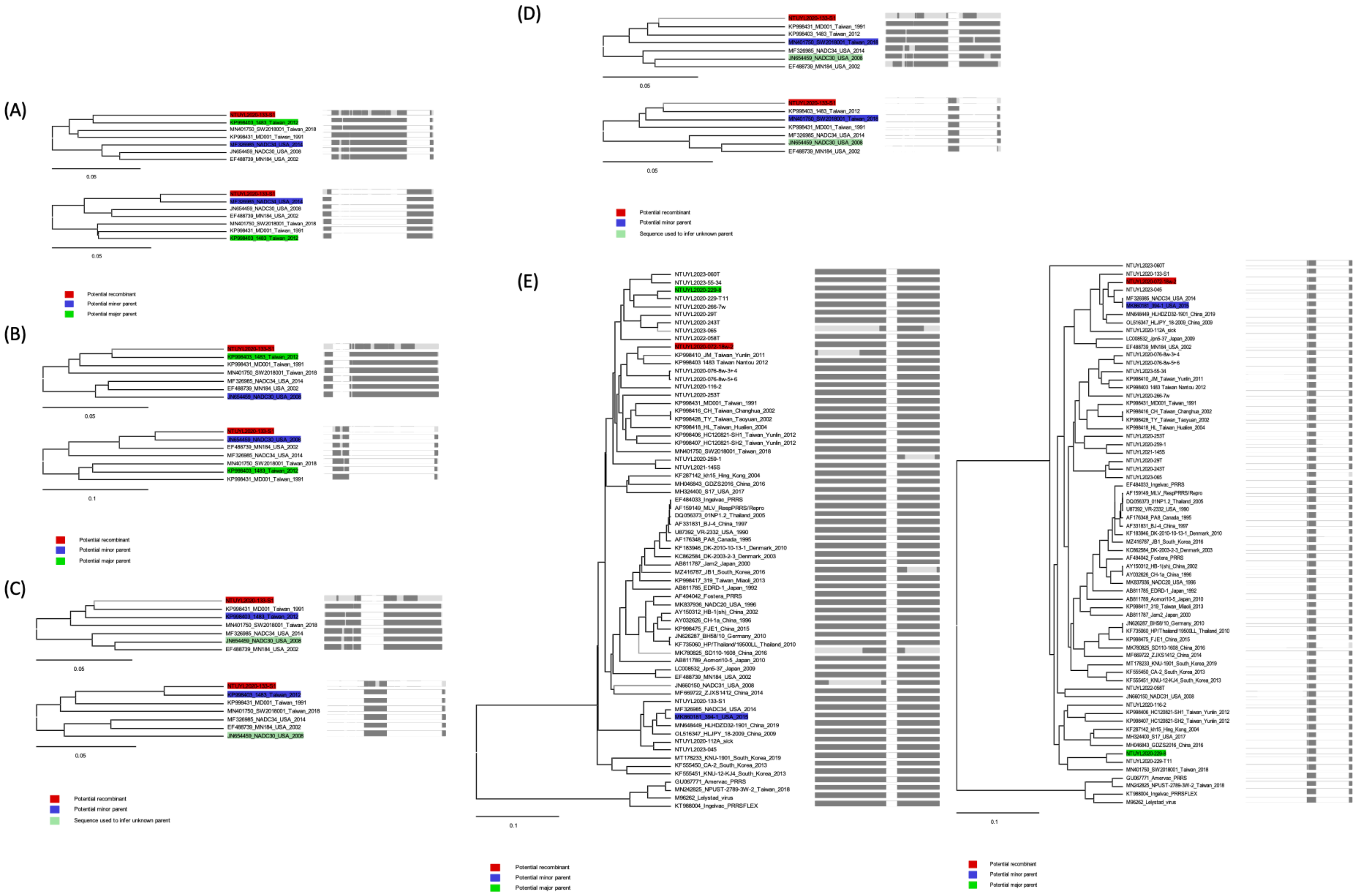

(A)
0.05
0.05
Potential recombinant
Potential minor parent
Potential major parent
(B)
0.05
0.1
Potential recombinant
Potential minor parent
Potential major parent
(C)
0.05
0.05
Potential recombinant
Potential minor parent
Sequence used to infer unknown parent
(D)
0.05
0.05
Potential recombinant
Potential minor parent
Sequence used to infer unknown parent
(E)
0.1
0.1
Potential recombinant
Potential minor parent
Potential major parent
Potential recombinant
Potential minor parent
Potential major parent

Supplemetary Figure S1. Trees for potential recombination events. (A) Based on the 4687-nucleotide from nucleotide position 1-1676/12248-15521, NTUYL2020-133-S1 was grouped with NADC34. (B) Based on the 2271-nucleotide from nucleotide position 1696-3966, NTUYL2020-133-S1 was grouped with NADC30. (C) Based on the 2985-nucleotide from nucleotide position 5274-8258, NTUYL2020-133-S1 was grouped with 1483. (D) Based on the 1548-nucleotide from nucleotide position 8910-10457, NTUYL2020-133-S1 was grouped closely with SW2018001. (E) Based on the 215-nucleotide sequence, NTUYL2020-072-18W-2 was grouped with 934-1.
